# Alpha8 Integrin (*Itga8*) Signalling Attenuates Chronic Renal Interstitial Fibrosis by Reducing Fibroblast Activation, Not by Interfering with Regulation of Cell Turnover

**DOI:** 10.1371/journal.pone.0150471

**Published:** 2016-03-03

**Authors:** Ines Marek, Till Lichtneger, Nada Cordasic, Karl F. Hilgers, Gudrun Volkert, Fabian Fahlbusch, Wolfgang Rascher, Andrea Hartner, Carlos Menendez-Castro

**Affiliations:** 1 Department of Pediatrics and Adolescent Medicine, University Hospital of Erlangen, Erlangen, Germany; 2 Department of Nephrology and Hypertension, University Hospital of Erlangen, Erlangen, Germany; INSERM, FRANCE

## Abstract

The α8 integrin (*Itga8*) chain contributes to the regulation of cell proliferation and apoptosis in renal glomerular cells. In unilateral ureteral obstruction *Itga8* is de novo expressed in the tubulointerstitium and a deficiency of *Itga8* results in more severe renal fibrosis after unilateral ureteral obstruction. We hypothesized that the increased tubulointerstitial damage after unilateral ureteral obstruction observed in mice deficient for *Itga8* is associated with altered tubulointerstitial cell turnover and apoptotic mechanisms resulting from the lack of *Itga8* in cells of the tubulointerstitium. Induction of unilateral ureteral obstruction was achieved by ligation of the right ureter in mice lacking *Itga8*. Unilateral ureteral obstruction increased proliferation and apoptosis rates of tubuloepithelial and interstitial cells, however, no differences were observed in the tubulointerstitium of mice lacking *Itga8* and wild type controls regarding fibroblast or proliferating cell numbers as well as markers of endoplasmic reticulum stress and apoptosis after unilateral ureteral obstruction. In contrast, unilateral ureteral obstruction in mice lacking *Itga8* led to more pronounced tubulointerstitial cell activation i.e. to the appearance of more phospho-SMAD2/3-positive cells and more α-smooth muscle actin-positive cells in the tubulointerstitium. Furthermore, a more severe macrophage and T-cell infiltration was observed in these animals compared to controls. Thus, *Itga8* seems to attenuate tubulointerstitial fibrosis in unilateral ureteral obstruction not via regulation of cell turnover, but via regulation of TGF-β signalling, fibroblast activation and/or immune cell infiltration.

## Introduction

Chronic renal fibrosis as a consequence of ureteral obstruction is characterized by complex changes in renal tissue homeostasis [1,2]. Increases in cell turnover and inflammatory infiltrates in the tubulointerstitium as well as altered interstitial deposition of matrix molecules and reduced extracellular matrix degradation are typical features of this disease [1,3]. In a murine model of unilateral ureter obstruction (UUO) induced by ligation of the ureter, the contribution of tubulointerstitial cell proliferation and apoptosis to the progression of renal fibrosis was extensively investigated. Renal fibroblasts and also tubular epithelial cells become highly proliferative [4]. Moreover, an increased apoptosis rate is observed in these cells [4] and inhibition of apoptosis protects against renal fibrosis after UUO [5]. Recently, increased endoplasmic reticulum (ER) stress resulting in increased apoptosis was described to be associated with the development of fibrosis in this model [6].

*Itga8* is a matrix receptor which is physiologically expressed on vascular smooth muscle cells and mesangial cells of the kidney [7]. In these cells it regulates cell attachment and migration [8,9]. Findings in cultivated vascular smooth muscle cells and cell lines suggest that *Itga8* might regulate cell growth and survival [10,11]. In murine UUO, *Itga8* is de novo expressed by tubulointerstitial fibroblasts and tubular epithelial cells [12]. We have previously shown that renal fibrosis after UUO is more severe in mice with a deficiency for the *Itga8* chain [12]. The mechanism by which *Itga8* expression attenuates the development of tubulointerstitial fibrosis is still unclear. A direct effect of *Itga8* expression on matrix turnover in several cell types could not be established [13]. Our own in vitro studies in glomerular mesangial cells, a cell type constitutively expressing *Itga8*, additionally argue for a role for *Itga8* signalling in regulating cell turnover: Expression of *Itga8* attenuated proliferation and apoptosis in these cells [8,14]. We therefore hypothesized that the de novo expression of *Itga8* in tubulointerstitial cells in UUO might attenuate renal fibrosis by reducing tubulointerstitial cell proliferation and/or apoptosis. We investigated if a lack of *Itga8* is associated with increased tubulointerstitial cell proliferation or apoptosis after UUO.

## Materials and Methods

### Animal procedures

As described previously [12], *Itga8* -deficient mice were from Dr. Ulrich Muller (Scripps Institute, LaJolla, USA) [15]. They were maintained on a mixed genetic background (C57BL/6x129Sv). They were used at the age of 10 weeks. Only male homozygous itga8-deficient mice (*Itga8* -/-) with two functional kidneys and an average weight of 20g were used for experiments. Age and weight matched male wild type (*Itga8* +/+) littermates served as controls.

To induce unilateral ureteral obstruction the right ureter was ligated. Eight *Itga8* 8+/+ and eight *Itga8* -/- were ligated and as controls eight *Itga8* +/+ and eight *Itga8*-/- were sham operated. Mice were sacrificed after seven days to isolate kidneys as described [12].

All procedures performed on animals were done in accordance with the NIH Guide for the Care and Use of Laboratory Animals and were approved by the local government authorities (Regierung von Mittelfranken, approval number AZ # 54–2532.1-17/08) after evaluation of the local government’s review board for animal research ethics. All surgery was performed under isoflurane or sodium pentobarbital anesthesia, and all efforts were made to minimize suffering. If judged necessary by a veterinarian, buprenorphine hydrochloride was injected to prevent or relief suspected pain or discomfort.

### Isolation of mRNA and real-time PCR

To study mRNA expression, total RNA was extracted with RNeasy^®^ Mini columns (Qiagen, Hilden, Germany) according to the manufacturer’s instructions. TaqMan reverse transcription reagents (Applied Biosystems, Weiterstadt, Germany) with random hexamers as primers were used to obtain first-strand cDNA. RNA concentration in the reverse transcription reaction mixture was adjusted to 100 ng/ μl. To test for genomic DNA contamination, reactions without Multiscribe reverse transcriptase were performed as negative controls. Real-time PCR was accomplished with an ABI PRISM 7000 Sequence Detector System and SYBR Green (Applied Biosystems) or TaqMan reagents (Applied Biosystems) according to the manufacturer’s protocol. The relative amount of the specific mRNA was normalized with respect to 18S rRNA. See S1 and S2 Tables) for primers and probes used for amplification. All samples were run in triplicates.

### Western blot analysis

Protein was isolated after mechanical homogenization of kidney tissue using a lysis buffer consisting of 50 mM Hepes pH 7.4, 1% Triton X-100, 150 mM NaCl, 1 mM EDTA, 10% Glycerol, 20 ml/ml proteinase inhibitor (1 tablet Complete proteinase inhibitor dissolved in 2 ml H_2_O, Santa Cruz Biotechnology, Heidelberg, Germany) and 2 mM sodium vanadate. Protein concentration was determined using a protein assay kit (Pierce, Rockford, IL, USA). For western blot analysis, 25μg protein was denatured before being separated on a 12% PAA gel (grp78), a 15% PAA gel (survivin) or 10% PAA gel (p-SMAD2/3). After electrophoresis, semi-dry blotting onto PVDF membranes (Pall Filtron, Karlstein, Germany) was performed. After blocking with Roti^®^-Block (Roth, Karlsruhe, Germany) membranes were incubated with the primary antibody. Immunoreactivity was visualized with a secondary horseradish peroxidase conjugated sheep anti-mouse or donkey anti-rabbit IgG antibody at a dilution of 1:10000 (both GE Healthcare, Munich, Germany) using the ECL-Plus system according to the manufacturer ‘s instructions (Pierce).

### Antibodies for Western blot analysis

The primary antibody to survivin (AF886; R&D Systems, Wiesbaden, Germany) was used at a dilution of 1:400, to grp-78 (ab21685; Abcam, Cambridge, UK) at a dilution of 1:200, to calnexin (ab75801; Abcam) at a dilution of 1:1500 and to p-SMAD2/3 (SC-11769; Santa Cruz) at a dilution of 1:5000. As a loading control tubulin, vinculin or amido black staining were used. The antibodies to tubulin or vinculin (both Sigma-Aldrich, Munich, Germany) were used at dilutions of 1:10000 (tubulin) or 1:5000 (vinculin).

### Immunostaining

For immunohistochemical staining kidneys were fixed in methyl carnoy’s solution and embedded in paraffin. 2μm sections were stained as described below.

For PCNA and survivin immunostaining kidney tissue was pretreated with TRS (DAKO, Hamburg, Germany). Sections were blocked with 3% H_2_O_2_. Primary antibodies were incubated overnight at the following dilutions: PCNA for proliferating cells (M0879; DAKO) 1:500, survivin (AF886; R&D Systems) 1:250, F4/80 for macrophages (LMU8949; Linaris, Dossenheim, Germany) 1:50, CD3 for T-cells (I7A2; BioLegend) 1:300, CD4 for T-helper cells (14-9766-82; eBioscience) 1:200, CD8a for cytotoxic T-cells (14-0808-82; eBioscience) 1:200, vimentin (GP53; Progen, Heidelberg, Germany) 1:50, p-SMAD2/3 (sc-11769; Santa Cruz Biotechnologies) 1:5000, α-smooth muscle actin (M 0851; DAKO) 1:50. Appropriate secondary antibodies (Vector, Burlingame, CA) were diluted 1:500, before avidin D peroxidase (Vector) was applied at a dilution of 1:2000. Finally DAB (Vector) was added, sections were counterstained with hematoxylin and covered with entellan.

### Apoptosis Assay

In situ detection of apoptosis was performed with TACS^®^ 2TdT-Blue Label in situ apoptosis detection kit (Trevigen, Biozol, Eching, Germany) according to the manufacturer’s protocol.

### Statistics

Analysis of variance (ANOVA), followed by post hoc Bonferroni test, was used to test significance of differences between groups. A p-value of less than 0.05 was considered significant. The procedures were carried out using the PASW Statistics 18 software (SPSS Inc., Chicago, USA). A student’s t-test was used to test significance of differences between two groups. Values are displayed as means±SEM.

## Results

### Parameters of cell turnover

**Fig 1:**
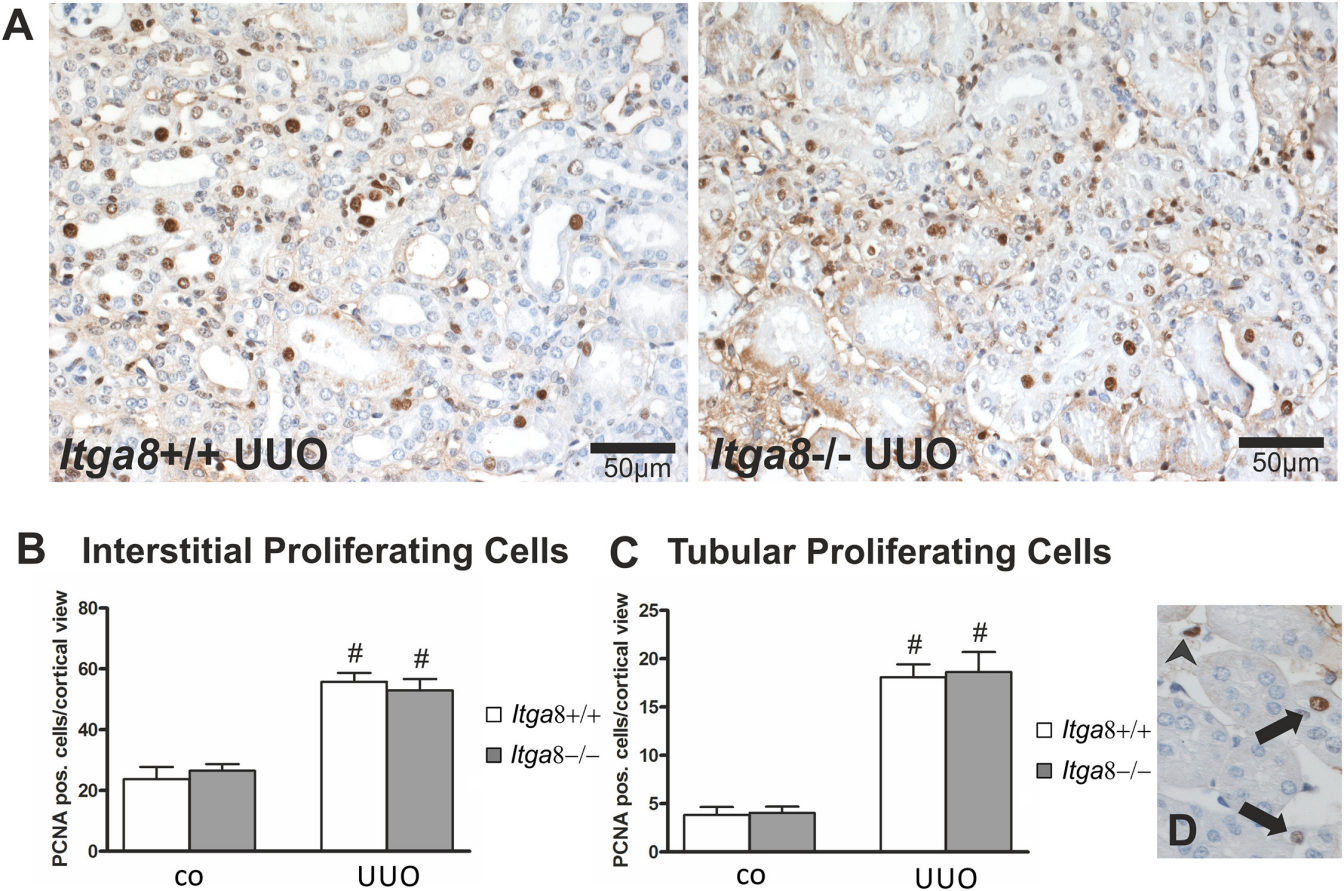
Cell proliferation in the renal tubulointerstitium after unilateral ureteral obstruction (UUO). A: Exemplary photomicrographs of UUO kidneys stained with PCNA. B: Counting of PCNA-positive interstitial cells. C: Counting of PCNA-positive tubuloepithelial cells. D: Photomicrograph showing PCNA-stained tubuloepithelial cells (black arrows) and PCNA-stained interstitial cells (black arrowhead). *Itga8*+/+, wild type mice; *Itga8*-/-, *Itga8*-deficient mice. Data are means±SEM. # p<0.05 UUO versus control (co).

**Fig 2:**
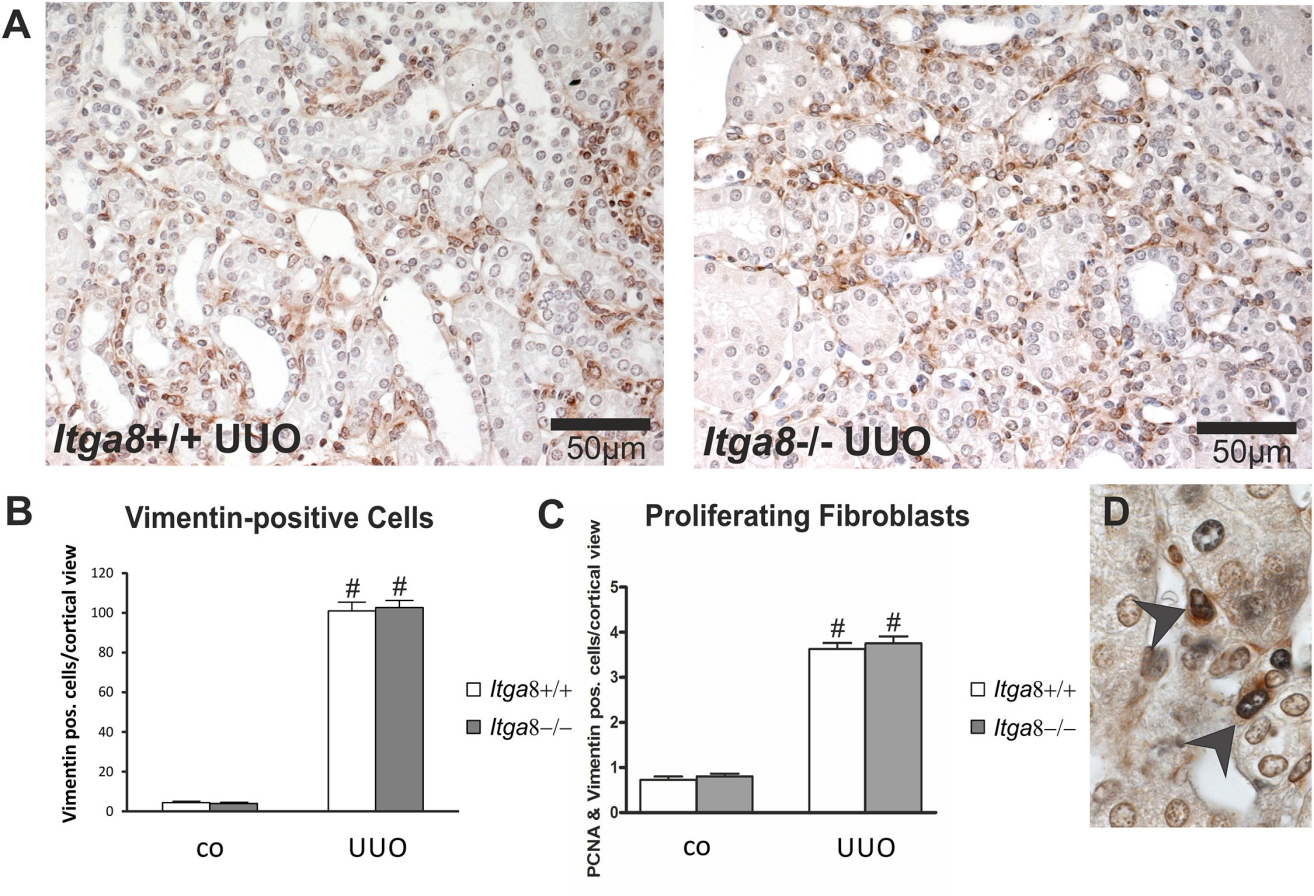
Fibroblast numbers in the renal interstitium after unilateral ureteral obstruction (UUO). A: Exemplary photomicrographs of UUO kidneys stained with vimentin. B: Counting of vimentin-positive interstitial cells. B: Counting of PCNA and vimentin double-stained interstitial cells. D: Photomicrograph showing PCNA (black) and vimentin (brown) costained cells (black arrowheads). *Itga8*+/+, wild type mice; *Itga8*-/-, *Itga8*-deficient mice. Data are means±SEM. # p<0.05 UUO versus control (co).

**Fig 3:**
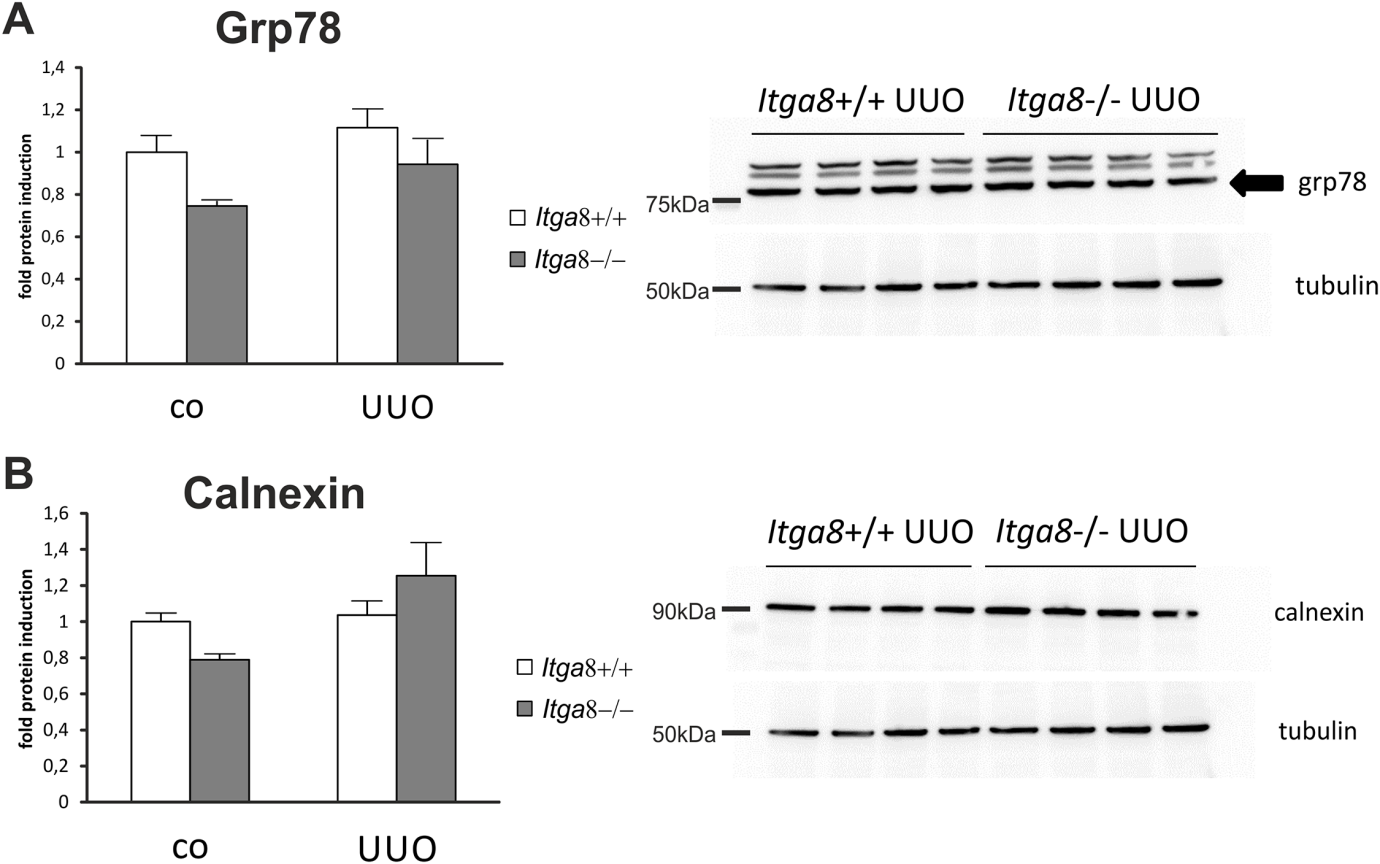
Markers of endoplasmic reticulum stress in the renal cortex. Western blot analysis of A, grp-78 and B, calnexin in the renal cortex of wild type and *Itga8*-/- mice after induction of unilateral ureter obstruction (UUO). Exemplary western blots are shown from the UUO groups. *Itga8*+/+, wild type mice; *Itga8*-/-, *Itga8*-deficient mice. Data are means±SEM.

**Fig 4:**
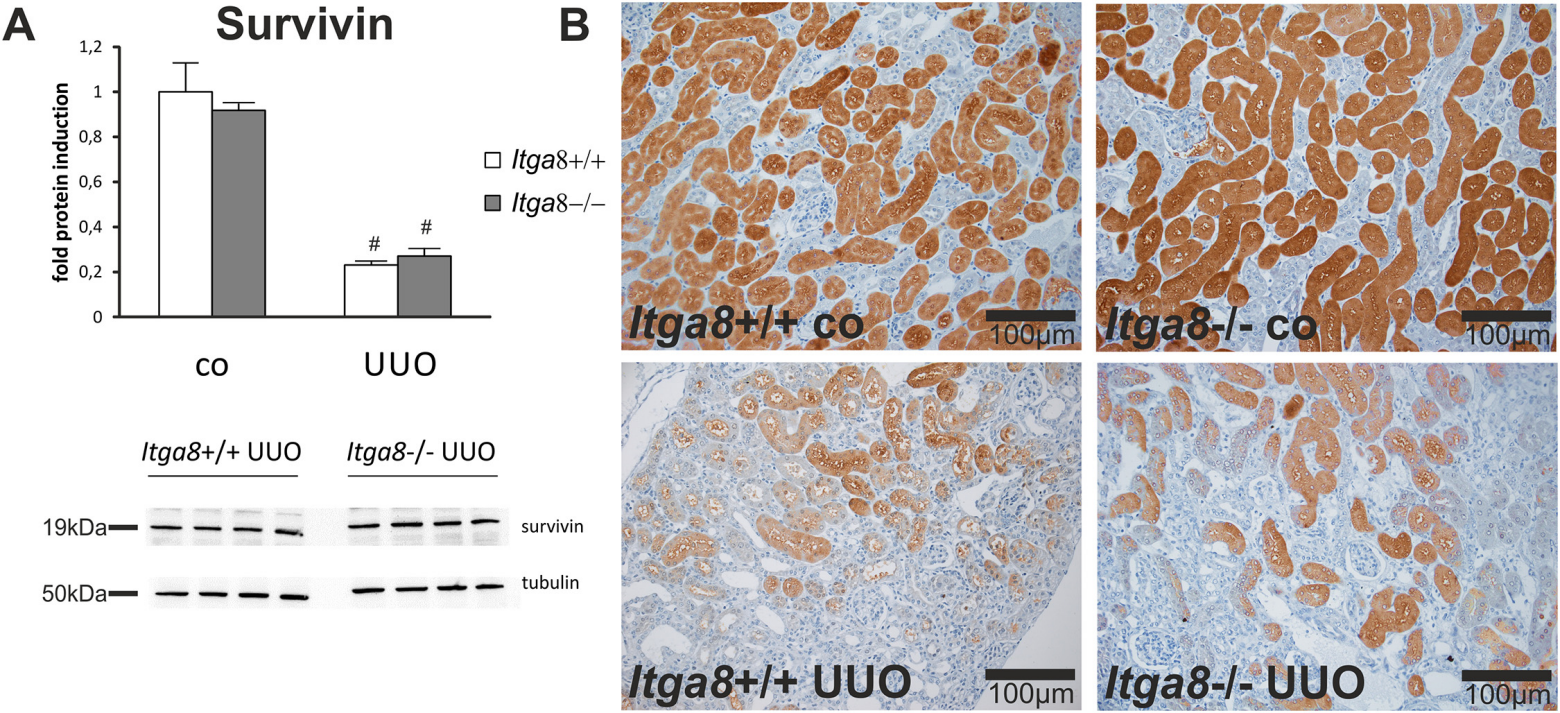
Survivin in tubulointerstitial cells. A, Western blot analysis of survivin protein in the renal cortex of wild type and *Itga8*-/- mice after induction of unilateral ureter obstruction (UUO). B,Exemplary photomicrographs show renal localization of survivin. *Itga8*+/+, wild type mice; *Itga8*-/-, *Itga8*-deficient mice. Data are means±SEM. # p<0.05 UUO versus control (co).

**Fig 5:**
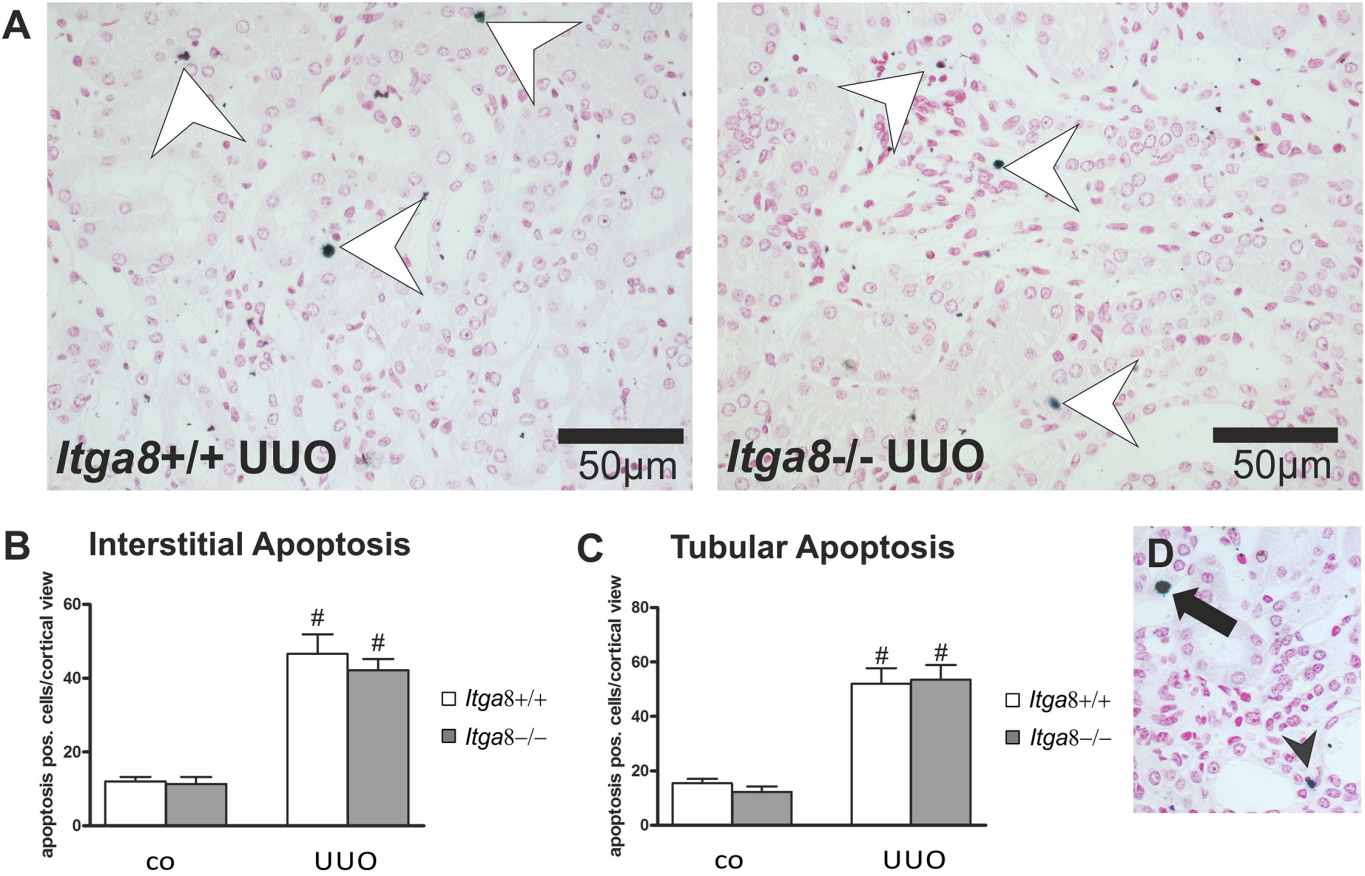
Apoptosis in tubulointerstitial cells. A, Exemplary photomicrographs show apoptotic cells in the tubulointerstitium (white arrowheads). B, Counting of apoptotic interstitial cells of wild type and *Itga8*-/- mice after induction of UUO. C, Counting of apoptotic tubuloepithelial cells of wild type and *Itga8*-/- mice after induction of UUO. D, Photomicrograph showing an apoptotic interstitial cell (black arrowhead) and an apoptotic tubuloepithelial cell (black arrow). *Itga8*+/+, wild type mice; *Itga8*-/-, *Itga8*-deficient mice. Data are means±SEM. # p<0.05 UUO versus control (co).

Tubuloepithelial and interstitial cell proliferation was investigated by staining for PCNA. In wildtype and *Itga8* -deficient control mice both the tubuloepithelial and the interstitial proliferation rate was comparably low (Fig 1). Induction of UUO resulted in an increase in PCNA-positive proliferating cells in both compartments, with no significant differences observed for wild type mice and *Itga8* -deficient mice (Fig 1). Counting of interstitial vimentin-positive mesenchymal cells revealed a strong increase in their numbers after UUO, however, no differences in wild types and *Itga8* -deficient mice were detected (Fig 2), arguing against significant differences in total fibroblast numbers. Counting of proliferating vimentin-positive fibroblasts also did not reveal differences in both genotypes (Fig 2). To assess the extent of ER stress, we evaluated markers of ER stress: *Atf4*, *Chop*, *Perk*, *Grp78* and *Calnexin*. *Perk* was very slightly induced in its expression after UUO, but was not different in both genotypes (Table 1). Neither *Atf4*, *Chop*, *Grp78* nor *Calnexin* mRNA expression was induced by UUO or *Itga8* deficiency (Table 1). Likewise, protein expression levels of grp78 and calnexin were not substantially different as assessed by western blot analyses (Fig 3A and 3B). Expression of p21, a marker of cell cycle arrest, was induced after UUO. Deficiency of *Itga8* did not result in changes in the expression of p21 in response to UUO (Table 1). The expression levels of the apoptosis markers bad, bax and bcl-2 were unchanged after UUO and not different in wild type and *Itga8*-deficient mice (Table 1). UUO led to an increase in p53 expression, which was comparable in both wild type and *Itga8*-deficient mice (Table 1). The expression of inhibitors of apoptosis, *Survivin*, *Ciap-1* and *Ciap-2*, but not of *Xiap*, was induced after UUO, however, it was not different in wild types and *Itga8*-deficient mice (Table 1). On the protein level, a reduction of survivin was observed after UUO by western blot analysis and immunohistochemistry (Fig 4), but again no differences were observed between wild types and *Itga8*-deficient mice. Evaluation of the numbers of apoptotic tubular and interstitial cells revealed a comparable increase of apoptotic cells in both compartments of wild types and *Itga8*-deficient mice after UUO (Fig 5).

**Table 1:** Expression analysis of parameters of cell turnover.

	*Itga8*+/+ co	*Itga8*-/- co	*Itga8*+/+ UUO	*Itga8*-/- UUO
*Grp78*	1.00±0.10	0.95±0.29	0.68±0.08	0.85±0.14
*Chop*,	1.00±0.32	0.36±0.03	0.72±0.09	0.76±0.07
*Perk*	1.00±0.14	0.84±0.08	1.59±0.16#	1.54±0.11#
*Atf4*	1.00±0.25	0.76±0.10	1.10±0.15	0.89±0.06
*Calnexin*	1.00±0.10	1.00±0.13	0.82±0.06	0.91±0.06
*P21*	1.00±0.15	0.60±0.20	2.90±0.31#	3.83±0.35#
*Bad*	1.00±0.05	0.90±0.13	0.75±0.09	0.90±0.13
*bax*	1.00±0.07	0.92±0.14	0.94±0.08	1.24±0.17
*bcl-2*	1.00±0.09	0.92±0.11	0.81±0.06	1.12±0.13
*P53*	1.00±0.11	1.08±0.14	1.85±0.19	2.24±0.31#
*Survivin*	1.00±0.16	1.04±0.17	4.98±0.84#	7.16±1.20#
*Xiap*	1.00±0.07	0.80±0.11	0.69±0.08	1.00±0.16
*Ciap-1*	1.01±0.07	0.80±0.12	2.21±0.24	3.1±0.50#
*Ciap-2*	1.00±0.11	0.79±0.14	6.24±0.53#	8.04±1.38#

Data are presented as fold induction (means±SEM).

^#^ p< 0.05 in unilateral ureter obstruction (UUO) versus control tissue (co). *Itga8*-deficient (*Itga8*-/-), wild type (*Itga8*+/+) mice.

### Interstitial cell activation and fibrosis

**Fig 6:**
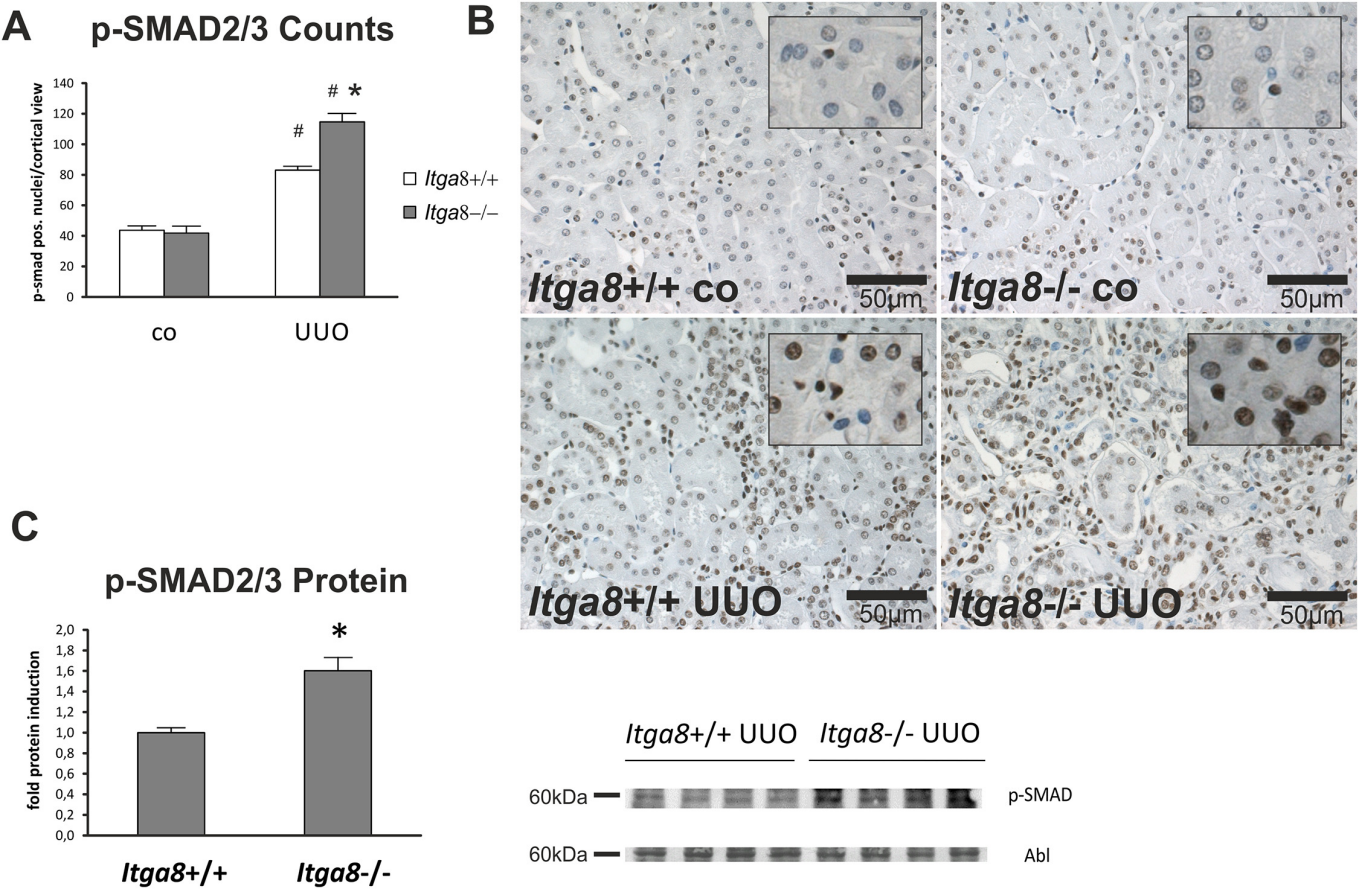
TGF-β signalling in the renal tubulointerstitium. A, Counting of p-SMAD2/3-positive nuclei in tubulointerstitial cells of wild type and *Itga8*-/- mice after induction of unilateral ureter obstruction (UUO). B, Exemplary photomicrographs of renal sections show staining for p-SMAD2/3. C, Western blot analysis comparing p-SMAD protein levels in both genotypes after UUO. *Itga8*+/+, wild type mice; *Itga8*-/-, *Itga8*-deficient mice. Data are means±SEM. # p<0.05 UUO versus control (co), * p<0.05 *Itga8*+/+ versus *Itga8*-/-.

**Fig 7:**
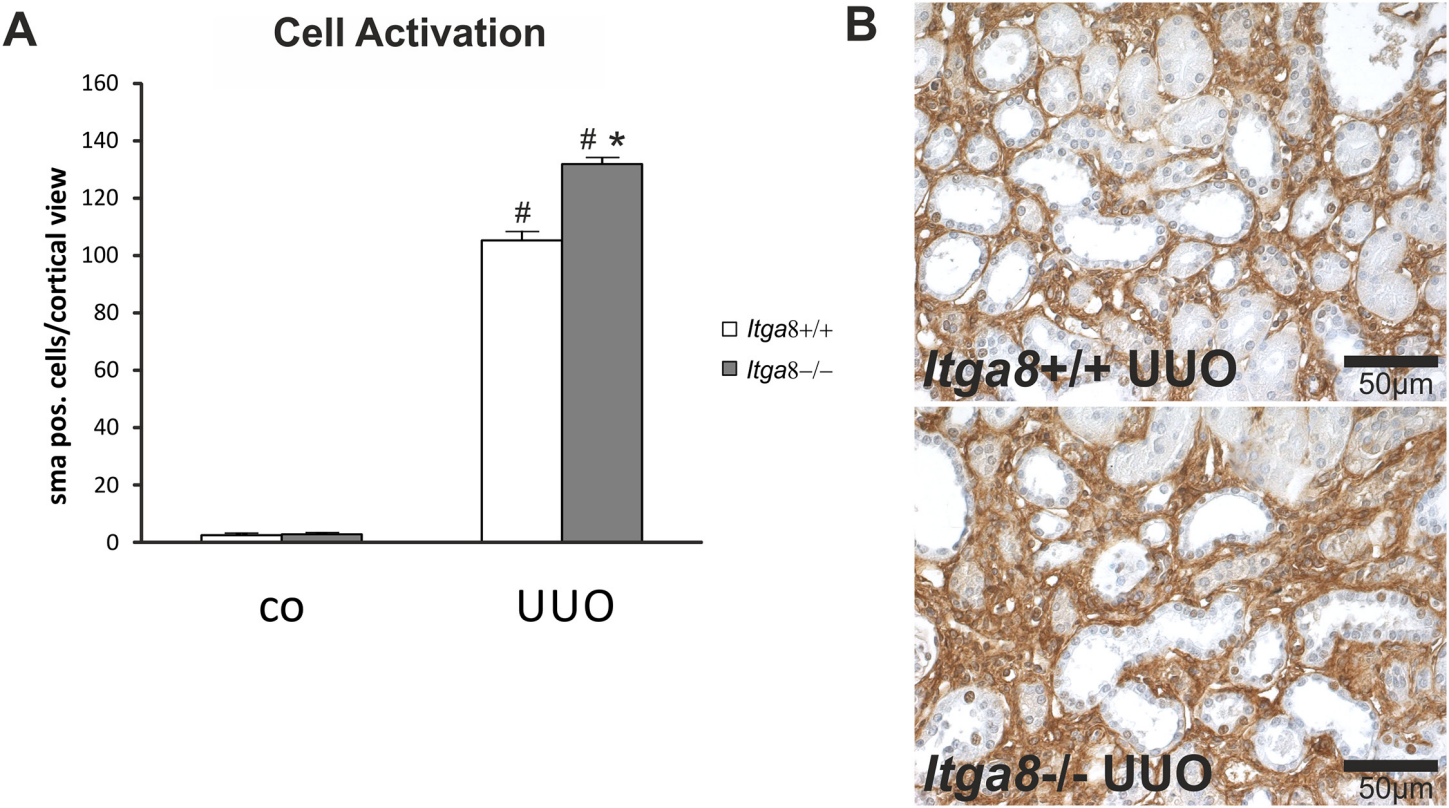
Cell activation in renal interstitial cells. A, Counting of α-smooth muscle actin-positive cells in the renal interstitium of wild type and *Itga8*-/- mice after induction of unilateral ureter obstruction (UUO). B, Exemplary photomicrographs of renal sections show staining for α-smooth muscle actin. *Itga8*+/+, wild type mice; *Itga8*-/-, *Itga8*-deficient mice. Data are means±SEM. # p<0.05 UUO versus control (co), * p<0.05 *Itga8*+/+ versus *Itga8*-/-.

As we did not detect differences in tubulointerstitial proliferation and apoptosis in wild-type and *Itga8*-deficient mice after UUO, we investigated TGF-β1-mediated cell activation and expression of fibrotic markers which are known to be secreted by activated fibroblasts. *Tgf-β1* expression was induced after UUO to a similar degree in wild types and *Itga8*-deficient kidneys (Table 2). UUO led to an upregulation of *Tgf-β2* expression, which tended to be more pronounced in *Itga8*-deficient mice compared to wild types (Table 2). The expression of TGF-β receptor 1 and 2 was induced in UUO, but was not different in wild types and *Itga8*-deficient kidneys (Table 2). Latent TGF-β binding protein 1 (*Ltbp-1*) expression was significantly increased by UUO only in *Itga8*-deficient mice (Table 2). TGF-β signalling was more markedly increased in *Itga8*-deficient mice compared to wild types after UUO, as assessed by western blot analysis for phospho-SMAD2/3 and counting of phospho-SMAD2/3-positive tubulointerstitial cells (Fig 6). The number of α-smooth muscle actin-positive interstitial cells was increased after UUO, but was higher in *Itga8*-deficient mice compared to wild types (Fig 7), confirming data from a previous study, detecting a more marked expansion of total interstitial α-smooth muscle actin staining by densitometric analysis [12]. Markers of matrix turnover *Mmp-2*, *Mmp-9*, *Timp-1 and Timp-2* were all increased in their expression levels after UUO, but were not different in wild types and *Itga8*-deficient kidneys (Table 2). Moreover, the expression of two molecules involved in matrix remodelling, i.e. PAI-1 and biglycan, was induced after UUO, but did not differ between wild type and *Itga8*-deficient mice (Table 2). As *Itga8* can interact with the PDGF-B pathway (Yabu J et al, Abstract at the 36th Annual Meeting of the American Society of Nephrology, 2003), we assessed the expression of *Pdgf-b* and its receptor *Pdgfrβ*. Both increased significantly after UUO, without any differences between wild type and *Itga8*-deficient mice (Table 2). VEGF-A and NO are vasoactive mediators and known to modulate tissue fibrosis. Thus, expression of *Vegf-a*, its receptor *Flt-1*, *Nos3* and *Nos2* was measured. The expression levels of *Vegf-a* and *Flt-1* were not altered by UUO, while expression of *Nos3* and *Nos2* was induced by UUO, but was not different in wild type and *Itga8*-deficient mice (Table 2).

**Table 2:** Expression analysis of regulators of fibrosis.

	*Itga8*+/+ co	*Itga8*-/- co	*Itga8*+/+ UUO	*Itga8*-/- UUO
*Tgf-β1*	1.00±0.13	0.75±0.13	6.47±0.64#	6.40±0.45#
*Tgf-β2*	1.00±0.14	0.80±0.15	7.02±1.36#	10.27±1.53#
*Ltbp-1*	1.01±0.13	0.73±0.08	1.46±0.10	1.60±0.19#
*Bmp-7*	1.00±0.08	1.04±0.12	0.37±0.05#	0.40±0.05#
*Tgf-βR1*	1.00±0.07	0.94±0.09	1.85±0.11#	1.91±0.19#
*Tgf-βR2*	1.00±0.13	1.03±0.15	3.29±0.10#	3.12±0.33#
*Biglycan*	1.00±0.06	0.85±0.14	6.65±0.49#	7.39±0.52#
*Pai-1*	1.00±0.45	0.40±0.08	10.78±3.11#	12.91±2.60#
*Mmp-2*	1.00±0.12	0.70±0.16	15.44±1.87#	17.08±1.95#
*Mmp-9*	1.00±0.21	0.75±0.33	3.99±0.71#	4.71±0.78#
*Timp-1*	1.01±0.20	0.98±0.33	237.50±39.54#	320.26±59.06#
*Timp-2*	1.00±0.07	0.76±0.11	4.87±0.29#	5.20±0.39#
*Pdgf-b*	1.00±0.12	0.93±0.15	5.03±0.67#	4.64±0.47#
*Pdgfrβ*	1.00±0.15	0.70±0.13	5.71±0.52#	6.15±0.55#
*Vegf-a*	1.00±0.13	0.88±0.07	0.62±0.05#	0.60±0.06
*Flt-1*	1.00±0.12	0.84±0.16	1.03±0.07	1.03±0.11
*Nos3*	1.00±0.11	0.97±0.11	1.67±0.13#	1.68±0.13#
*Nos2*	1.00±0.17	0.80±0.15	5.03±0.57#	5.80±1.08#

Data are presented as fold induction (means±SEM).

^#^ p< 0.05 in unilateral ureter obstruction (UUO) versus respective control tissue (co). *Itga8*-deficient (*Itga8*-/-), wild type (*Itga8*+/+) mice.

### Interstitial inflammation

**Fig 8:**
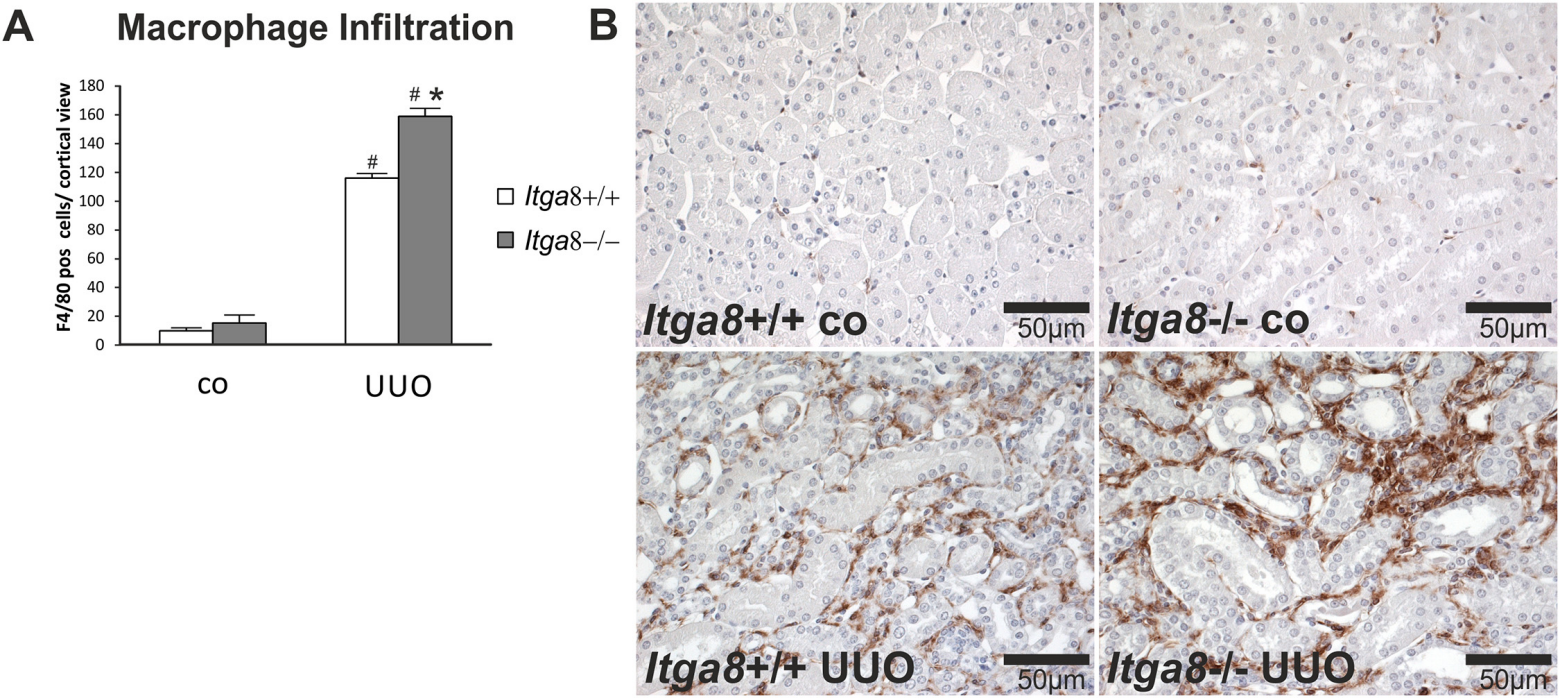
Macrophage infiltration in the renal interstitium. A, Counting of F4/80-positive cells in the renal interstitium of wild type and *Itga8*-/- mice after induction of unilateral ureter obstruction (UUO). B, Exemplary photomicrographs of renal sections show staining for F4/80. *Itga8*+/+, wild type mice; *Itga8*-/-, *Itga8*-deficient mice. Data are means±SEM. # p<0.05 UUO versus control (co), * p<0.05 *Itga8*+/+ versus *Itga8*-/-.

**Fig 9:**
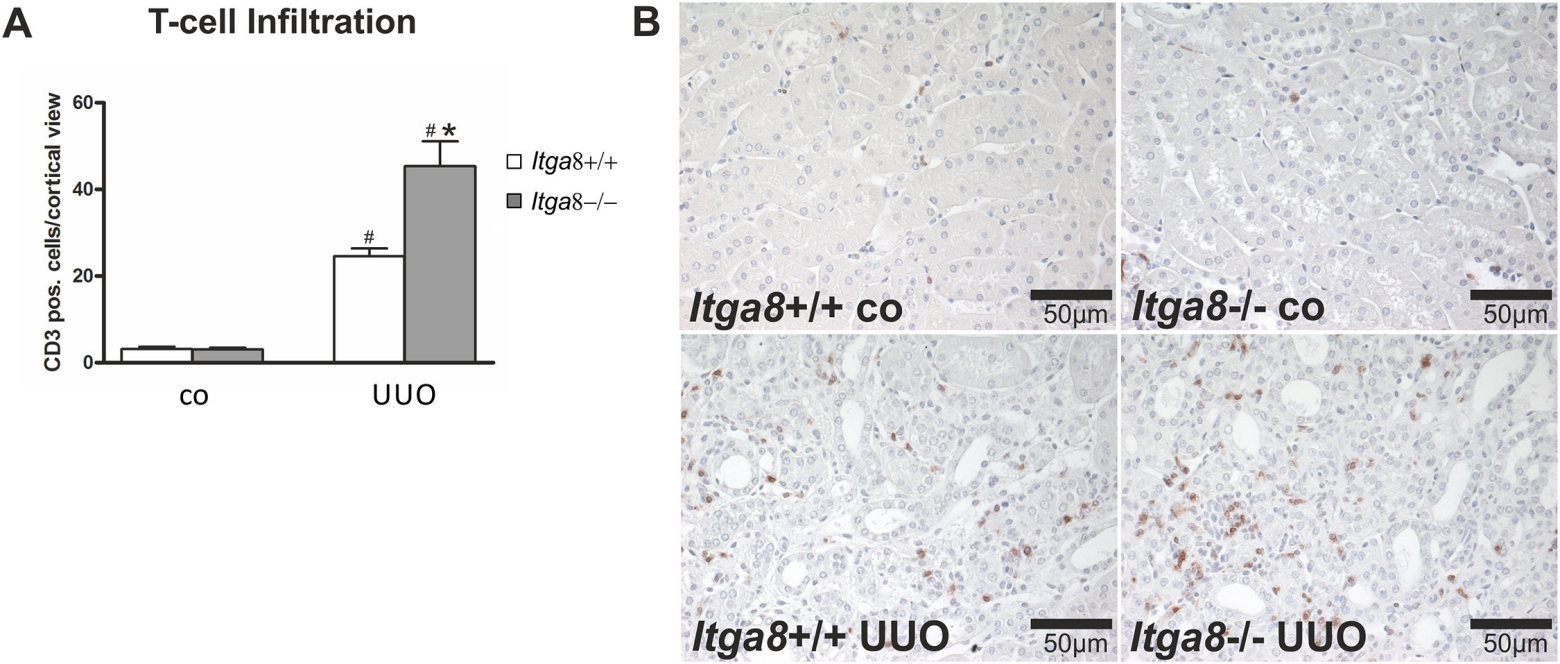
T-cell infiltration in the renal interstitium. A, Counting of CD3-positive cells in the renal interstitium of wild type and *Itga8*-/- mice after induction of unilateral ureter obstruction (UUO). B, Exemplary photomicrographs of renal sections show staining for CD3. *Itga8*+/+, wild type mice; *Itga8*-/-, *Itga8*-deficient mice. Data are means±SEM. # p<0.05 UUO versus control (co), * p<0.05 *Itga8*+/+ versus *Itga8*-/-.

**Fig 10:**
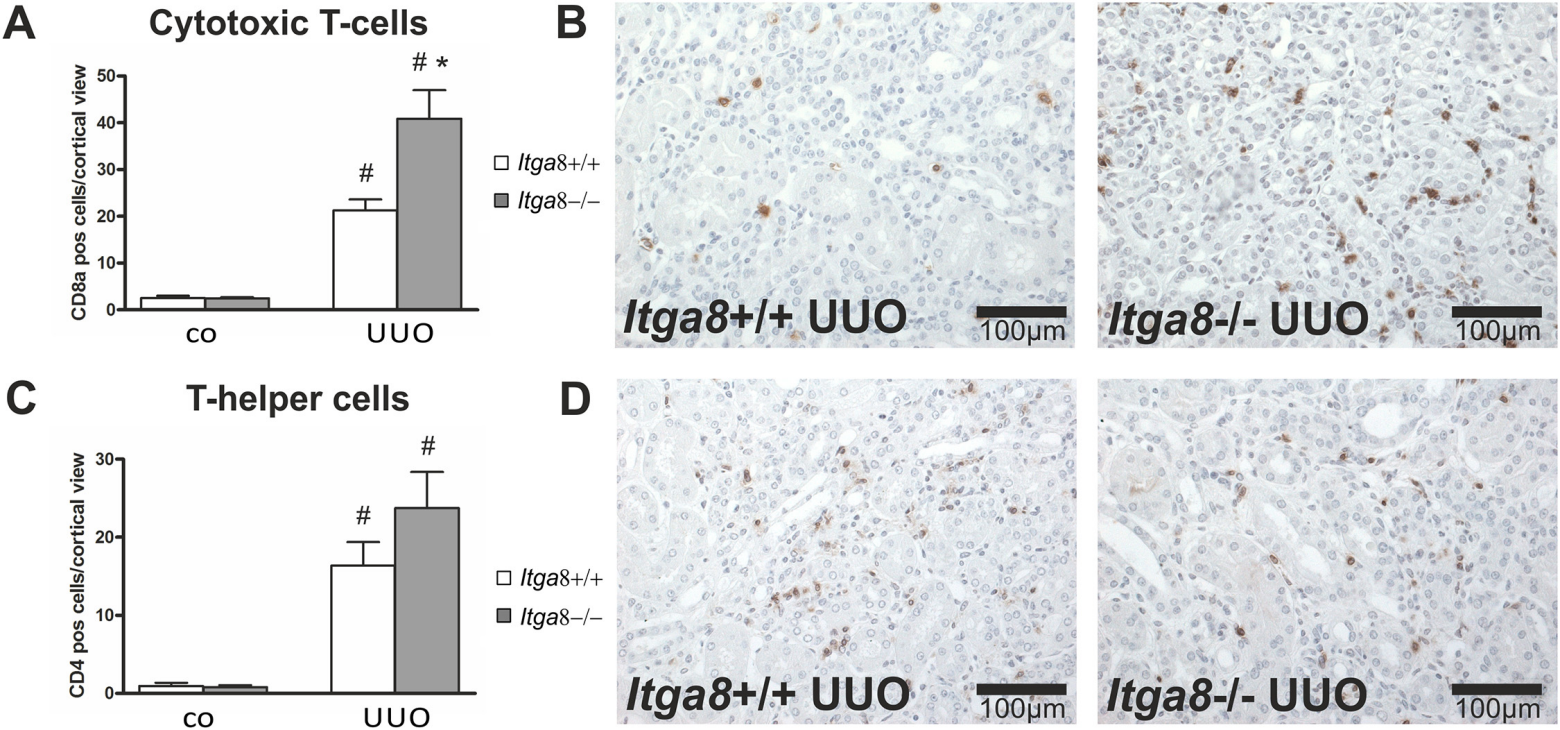
Infiltration of T-cell subsets in the renal interstitium. A, Counting of CD8a-positive cytotoxic T-cells in the renal interstitium of wild type and *Itga8*-/- mice after induction of unilateral ureter obstruction (UUO). B, Exemplary photomicrographs of renal sections show staining for CD8a. C, Counting of CD4-positive T-helper cells in the renal interstitium of wild type and *Itga8*-/- mice after induction of unilateral ureter obstruction (UUO). D, Exemplary photomicrographs of renal sections show staining for CD4. *Itga8*+/+, wild type mice; *Itga8*-/-, *Itga8*-deficient mice. Data are means±SEM. # p<0.05 UUO versus control (co), * p<0.05 *Itga8*+/+ versus *Itga8*-/-.

Activation of interstitial fibroblasts in UUO commonly results in secretion of chemoattractants which promote the infiltration of immune cells, mainly macrophages and T-cells. Thus, we studied interstitial inflammation. Investigation of the expression levels of proinflammatory cytokines *Tnf-α*, *Il-β and Il-6*, the chemokines *Mcp-1*, Rantes and *Cxcl3*, the proinflammatory proteins S100A8 and S100A9, as well as adhesion molecules ICAM-1 and VCAM revealed an induction after UUO (Table 3). Moreover, macrophage, total T-cell, cytotoxic T-cell and T-helper cell infiltration was increased after UUO (Figs 8, 9 and 10). While macrophage infiltration as well as total and cytotoxic T-cell infiltration into the interstitium was significantly higher in *Itga8*-deficient kidneys compared to wild types (Figs 8, 9 and 10), the expression of cytokines (*Il-1β*, *Il-6*, *Tnf-α)*, chemokines (*Mcp-1*, *Rantes*, *Cxcl3*) or adhesion molecules (*Icam-1*, *Vcam*) investigated was clearly induced by UUO, but did not differ between wild type and *Itga8*-deficient kidneys (Table 3). S100A8 and S100A9 form a heterodimeric protein complex called calprotectin. They are released during activation of phagocytes and amplify inflammatory responses [16]. Both proteins were induced in UUO, but again, no differences were detected between wild type and *Itga8*-deficient kidneys (Table 3).

**Table 3:** Expression analysis of mediators of inflammation.

	*Itga8*+/+ co	*Itga8*-/- co	*Itga8*+/+ UUO	*Itga8*-/- UUO
*Il-1*	1.00±0.29	0.50±0.22	2.86±0.62#	2.31±0.15#
*Il-6*	1.00±0.29	0.59±0.22	82.77±29.21	101.58±22.34#
*Tnf-α*	1.00±0.36	0.42±0.04	8.61±0.96#	9.04±0.59#
*Mcp-1*	1.00±0.22	0.76±0.06	14.70±1.50#	12.29±1.51#
*Rantes*	1.00±0.16	1.00±0.15	33.52±4.24#	26.32±3.75#
*Cxcl3*	1.00±0.30	0.41±0.14	21.43±3.64#	17.56±2.31#
*S100A8*	1.00±0.31	0.90±0.38	2.64±0.32#	2.34±0.59
*S100A9*	1.00±0.25	0.94±0.37	3.51±0.50#	3.06±0.60#
*Icam-1*	1.00±0.10	1.08±0.14	9.36±0.48#	8.91±0.72#
*Vcam*	1.00±0.12	0.95±0.18	78.54±3.92#	81.46±9.84#

Data are presented as fold induction (means±SEM).

^#^ p< 0.05 in unilateral ureter obstruction (UUO) versus respective control tissue (co). *Itga8*-deficient (*Itga8*-/-), wild type (*Itga8*+/+) mice.

## Discussion

Unilateral ureter obstruction (UUO) leads to changes in renal cell and matrix turnover and finally induces tubulointerstitial fibrosis [2]. In previous studies we detected a de novo expression of *Itga8* in the tubulointerstitium after UUO and found a more severe interstitial fibrosis in *Itga8*-deficient mice compared to wild type litters [12]. The aim of the present study was to assess the hypothesis that *Itga8* might serve a protective function in tubulointerstitial cells by reducing the increased proliferation and apoptosis rates after UUO. Taken together, our findings confirm that proliferation and apoptosis are upregulated in UUO, but we did not observe any effects of *Itga8* expression in tubulointerstitial cells on proliferation, interstitial cell number or tubulointerstitial apoptosis rates, which might explain the more severe disease progression seen in *Itga8*-deficient mice. This is in contrast to findings in mesangial cells, which constitutively express *Itga8*. In *Itga8*-deficient mice, some degree of mesangial hyperplasia exists [17]. Induction of a mesangioproliferative glomerulonephritis in these mice results in prolonged proliferation and apoptosis in the glomerulus [14]. Moreover, in vitro studies revealed a role for *Itga8* signalling in reducing mesangial cell proliferation and apoptosis [8,14]. Thus, the contribution of *Itga8* signalling to the regulation of cell turnover seems to be cell type dependent. Although the inhibitor of apoptosis survivin was found to be upregulated in cultivated *Itga8*-deficient cells compared to wild type cells (unpublished observations), there was no difference in renal survivin expression or protein abundance in wild type and *Itga8*-deficient mice, further arguing against a contribution of *Itga8* to the regulation of cell survival in UUO. Surprisingly, the mRNA expression of survivin was increased, but survivin protein was reduced by UUO, irrespective of the mouse genotype. This might be due to the fact that survivin protein is primarily regulated via tubular uptake from the primary urine and not via expression by renal cells [18].

As mediators of ER stress can be regulated by integrin signalling [19], we also studied the influence of *Itga8* expression on markers of ER stress. Unlike in a study in Wistar rats, where ER stress markers were induced by UUO [6], we did not detect an induction of ER stress after UUO in our mouse model. This discrepancy might be explained by species differences. Moreover, our findings suggest that *Itga8* signalling is not necessary for regulating the expression of ER stress markers. With sustained ER stress commonly leading to apoptosis, these findings also do not support the notion that *Itga8* signalling might attenuate apoptosis in tubulointerstitial cells after UUO.

Why then does a lack of *Itga8* aggravate renal fibrosis after UUO? Findings from previous studies suggest that *Itga8* expression might have an influence on cell phenotype [20] and might regulate the degree of fibroblast or mesangial cell activation [14,21,22]. In mice deficient for *Itga8* glomerular mesangial cells are more frequently positive for α-smooth muscle actin than mesangial cells in wild type mice [21]. This was also observed in fibroblasts in our model of renal fibrosis: Interstitial cells of *Itga8*-deficient kidneys after UUO are more frequently positive for α-smooth muscle actin than interstitial cells from wild types. On the other hand, there was no difference in the number of vimentin-positive fibroblasts, indicating that the total number of fibroblasts is not different in *Itga8*-deficient and wild type mice after UUO. Thus, it might be conceivable that *Itga8*-deficient fibroblasts acquire a different state of activation compared to wild type renal fibroblasts during tubulointerstitial fibrosis.

As TGF-β signalling decisively contributes to fibrosis by regulating matrix proteases and their inhibitors as well as inducing extracellular matrix production [2,23–25], we investigated *Tgf-β1* and *Tgf-β2* expression levels. *Tgf-β1* expression was induced after UUO, but not different in *Itga8*-deficient and wild type mice. The induction of *Tgf-β2* expression after UUO tended to be more marked in *Itga8*-deficient mice, without reaching statistical significance. TGF-beta receptor expression did not differ in the kidneys of *Itga8*-deficient and wild type mice. *Bmp-7*,a negative regulator of TGF-β signalling [26], was reduced in its expression after UUO in both *Itga8*-deficient and wild type mice to a similar degree. However, *Ltbp-1* which is known to promote TGF-β activity [27,28], was significantly upregulated after UUO in *Itga8-*deficient mice only. As TGF-β action is often regulated via activation of latent TGF-β and not via expressional changes [29,30], we further studied the TGF-β pathway. TGF-β signalling, as assessed by phospho-SMAD2/3 levels and SMAD2/3 translocation into the nucleus [31–33] was more increased in *Itga8*-deficient mice compared to wild types after UUO. This might be due to an inhibitory action of itga8 which was shown to bind to the latent form of TGF-β and thereby inhibiting TGF-β activation [34]. We speculate that in *Itga8*-deficient mice TGF-β signalling is induced by activation of former inactive TGF-β [35], possibly supported by LTBP-1, and therefore is not dependent on *Tgf-β* expression. This might lead to the increased deposition of collagen I in *Itga8*-deficient mice which was observed after UUO [12]. Several regulators of extracellular matrix turnover, *Mmp-2*, *Mmp-9*, *Timp-1 and Timp-2*, were upregulated by UUO, but not different in *Itga8*-deficient and wild type mice and are therefore not likely to contribute to the increased interstitial expansion of collagen I in *Itga8*-deficient mice. The same holds true for other known regulators of extracellular matrix accumulation in the tubulointerstitium, like PAI-1, PDGF-B, VEGF-A, Biglycan and NO [36–39].

As activated fibroblasts may play a role as modulators of renal inflammatory processes, which are commonly observed after UUO [2,40], we then quantified leukocyte infiltration. The leukocytes, which infiltrate the kidney after UUO mainly consist of macrophages and T-cells [4]. Mice with a deficiency for *Itga8* show more macrophages and more T-cells infiltrating the kidney after UUO than wild type mice, which might contribute to the more severe renal fibrosis in these mice. On the other hand, typical mediators of inflammation, like the cytokines *Tnf-α*, *Il-1β* and *Il-6*, the adhesion molecules *Vcam* and *Icam-1* as well as the chemokines *Mcp-1*, *Cxcl3* and *Rantes* are upregulated by UUO [41], but not different in *Itga8*-deficient and wild type mice. Thus, it remains unclear, which inflammatory mediators might account for the increased leukocyte infiltration in *Itga8*-deficient mice. Our findings do not completely clarify the pathways leading to increased fibrosis in *Itga8*-deficient mice. We detected increased TGF-*β* signalling, an increased amount of activated fibroblasts and increased immune cell infiltration. Basically, activation of fibroblasts might lead to macrophage and T-cell infiltration, or, alternatively, the increased number of immune cells might result in fibroblast activation [42–45], or both phenomena might be triggered by an unknown factor. After UUO a direct association between the amounts of activated fibroblasts and infiltrating macrophages was described [46]. The study is limited in that our observations are only associations, but a functional link was not established. Moreover, renal mass is reduced about 20–30 percent in *Itga8*-deficient mice [12,17] which might affect the phenotype. Because of developmental defects in these mice [15] we cannot completely rule out that pelvis structure or compliance might be different in itga8-deficient mice which might contribute to the more severe outcome after UUO.

Taken together our findings suggest that the increased renal fibrosis detected in *Itga8*-deficient mice is not a consequence of an augmented tubulointerstitial cell turnover, but more likely due to a higher degree of fibroblast activation and/or to more macrophage and T-cell infiltration.

## Supporting Information

S1 Table Primers used for Real-time PCR analysis. (DOC)

S2 Table Mouse primers forward (fw), reverse (rv) and probes (taq) for TaqMan-PCR. (DOCX)
