# Supplementary material for: Alpha8 Integrin (Itga8) Signalling Attenuates Chronic Renal Interstitial Fibrosis by Reducing Fibroblast Activation, Not by Interfering with Regulation of Cell Turnover
Source: PLoS One. 2016 Mar 3;11(3):e0150471. doi: 10.1371/journal.pone.0150471 (PMC4777439; doi:10.1371/journal.pone.0150471)
Supplement: S1 Table — (DOC) [file pone.0150471.s001.doc]

**S1 Table: Primers used for Real-time PCR analysis**

| **mouse primer** | **forward** | **reverse** |
| --- | --- | --- |
| ***Atf4*** | 5‘-CATGGCGCTCTTCACGAAAT-3‘ | 5‘-TCGCTGTTCAGGAAGCTCATC-3‘ |
| ***Bad*** | 5‘-CGAAGGATGAGCGATGAGTTT-3‘ | 5‘-TTTGTCGCATCTGTGTTGCA-3‘ |
| ***Bax*** | 5‘-AGACACCTGAGCTGACCTTGGA-3‘ | 5‘-GGAGACACTCGCTCAGCTTCTT-3‘ |
| *Bcl2* | 5‘-CCTGCATTTAGCAAGCTGCTT-3‘ | 5‘-GGGCCAACGCGCTTTC-3‘ |
| ***Biglycan*** | 5‘-ACATTTCTGAGCTTCGCAAGG-3‘ | 5‘-CGTAGAGGTGCTGGAGGCC-3‘ |
| ***Bmp-7*** | 5‘-CAGGGCCCCCCTTTAGC-3‘ | 5‘-TGTTCCACTAGGTTGACGAAGCT-3‘ |
| ***Calnexin*** | 5‘-GAATGTGGTGGTGCCTATGTGA-3‘ | 5‘-GGAGTCTTGTCGTGGAATTGATC-3‘ |
| ***Chop*** | 5‘-GAAGAGGAAGAATCAAAAACCTTCA-3‘ | 5‘-ATGTGCGTGTGACCTCTGTTG-3‘ |
| ***Ciap-1*** | 5‘-TGTGATGGTGGCTTGAGATGTT-3‘ | 5‘-CGTATCAAGAACTCACACCTTGGA-3‘ |
| ***Ciap-2*** | 5‘-TTGAGCAGCTATTATCTACGTCAGACT-3‘ | 5‘-TTTAACCACAGGCGTGCTCAT-3’ |
| **Cxcl-3** | 5‘-TTTGAGACCATCCAGAGCTTGA-3‘ | 5‘-CCTTGAGAGTGGCTATGACTTCTGT-3’ |
| *Grp78* | 5‘-GCCTCATCGGACGCACTT-3‘ | 5‘-AACCACCTTGAATGGCAAGAA-3‘ |
| ***Icam-1*** | 5‘-GCATTGTTCTCTAATGTCTCCGAG-3‘ | 5‘-AAGATCGAAAGTCCGGAGGC-3‘ |
| ** *Il-1β*** | 5‘-GCCACCTTTTGACAGTGATGAG-3‘ | 5‘-TCTTTTGGGGTCCGTCAACT-3‘ |
| ***Il-6*** | 5‘-CCACGGCCTTCCCTACTTC-3‘ | 5‘-TGCACAACTCTTTTCTCATTTCCA-3‘ |
| ***Ltbp-1*** | 5‘-CCCCAAGAAACAATCCTATCATG-3‘ | 5‘-TGTAGGCATTCCATCATTTGAGTATATC-3‘ |
| ***Mmp2*** | 5‘-ATGCGGAAGCCAAGATGTG-3‘ | 5‘-GTCCAGGTCAGGTGTGTAAC-3‘ |
| ***Mmp9*** | 5‘-CAAGGATTGCTCAGAGATTCTCCG-3‘ | 5‘-ATCTCACCTGGAGGACACAGTCTG-3‘ |
| ***Nos2*** | 5‘-ACCCCTGTGTTCCACCAGGAGATGTTGAA-3‘ | 5‘-TGAAGCCATGACCTTTCGCATTAGCATGG-3‘ |
| *Nos3* | 5‘-CACCAGGAAGAAGACCTTTAAGGA-3‘ | 5‘-CACCGTGCCCATGAGTGA-3‘ |
| ***P53*** | 5‘-CACAGCGTGGTGGTACCTTATG-3‘ | 5‘-TGTACTTGTAGTGGATGGTGGTATACTCA-3‘ |
| *P21* | 5‘-TTCGGTCCCGTGGACAGT-3‘ | 5‘-CATGAGCGCATCGCAATC-3‘ |
| ***Perk*** | 5‘-CCCACAGGCAGCGGAAG-3‘ | 5‘-GTCACTGACATCGGCACTCA-3‘ |
| ***Rantes*** | 5‘-GTGCCCACGTCAAGGAGTAT-3‘ | 5‘-GAGTGACAAACACGACTGCAA-3‘ |
| ***Survivin*** | 5‘-CAACCCGATAGAGGAGCATAGAAA-3‘ | 5‘-TTCCATCTGCTTCTTGACAGTGA-3‘ |
| ***S100A8*** | 5‘-TCCTTTGTCAGCTCCGTCTTC-3‘ | 5‘-ACGAAAGATTTCCTTTCAAACGATG-3‘ |
| ***S100A9*** | 5‘-CTCAGATGGAGCGCAGCATA-3‘ | 5‘-CTCTTTCTTCATAAAGGTTGCCAACT-3‘ |
| ***Tgf-βR1*** | 5‘-CAGGACCACTGCAATAAAATAGAACT-3‘ | 5‘-AGGACCAAGGCCAGCTGACT-3‘ |
| ***Tgf-βR2*** | 5‘-CGGGCGAGACTTTCTTCATG-3‘ | 5‘-ACACCCGTCACTTGGATAATGAC-3‘ |
| ***Tgf-β 2*** | 5‘-CTGTACCTTCGTGCCGTCTAATAA-3‘ | 5‘-TGCCATCAATACCTGCAAATCT-3‘ |
| ***Timp1*** | 5‘-GATATGTCCACAAGTCCCAGAACC-3‘ | 5‘-CCACAGCCAGCACTATAGGTCTTT-3‘ |
| ***Timp2*** | 5‘-ATAAAGATGTTCAAAGGACCTGACAA-3‘ | 5‘-GGCCGTGTAGATAAACTCGATGT-3‘ |
| ***Tnf-α*** | 5‘-AGGGATGAGAAGTTCCCAAATG-3‘ | 5‘-TCTGGGCCATAGAACTGATGAGA-3‘ |
| ***Vcam*** | 5‘-CTACAAGTCTACATCTCTCCCAGGAA-3‘ | 5‘-GGAGGGATGTACAGAGATCGTTG-3‘ |
| ***Vegf-a*** | 5‘-AACGAAAGCGCAAGAAATCC-3‘ | 5‘-GCTCACAGTGAACGCTCCAG-3‘ |
| ***Xiap*** | 5‘-CGGATCGTTACTTTTGGAACATG-3‘ | 5‘-CGCCTTCACCTAAAGCATAAAATC-3‘ |
| **18S rRNA** | 5‘-TTGATTAAGTCCCTGCCCTTTGT-3‘ | 5‘-CGATCCGAGGGCCTCACTA-3‘ |
