# Supplementary material for: Alpha8 Integrin (Itga8) Signalling Attenuates Chronic Renal Interstitial Fibrosis by Reducing Fibroblast Activation, Not by Interfering with Regulation of Cell Turnover
Source: PLoS One. 2016 Mar 3;11(3):e0150471. doi: 10.1371/journal.pone.0150471 (PMC4777439; doi:10.1371/journal.pone.0150471)
Supplement: S2 Table — (DOCX) [file pone.0150471.s002.docx]

**S2 Table: Mouse primers forward (fw), reverse (rv) and probes (taq) for TaqMan-PCR**

| ***Flt-1*** | **taq** | 5‘-CGCTCCTCCCAAAATGAGTTTGGACAT-3‘ |
| --- | --- | --- |
|  | **fw** | 5‘-CTAACTTTCAGGCCCAGAGGA-3‘ |
|  | **rv** | 5‘-GGCACTGGGCTTTCTTATTAC-3‘ |
| ***Mcp1*** | **taq** | 5‘-CACCAGCAAGATGATCCCAATGAGTAGGC-3‘ |
|  | **fw** | 5‘-CCACTCACCTGCTGCTACTCAT-3‘ |
|  | **rv** | 5‘-TGGTGATCCTCTTGTAGCTCTCC-3‘ |
| ***Tgf-β1*** | **taq** | 5‘-TTCAGCGCTCACTGCTCTTGTGACAG-3‘ |
|  | **fw** | 5‘-TGACGTCACTGGAGTTGTACGG-3‘ |
|  | **rv** | 5‘-GGTTCATGTCATGGATGGTGC-3‘ |
